# Social Class Differences in Secular Trends in Established Coronary Risk Factors over 20 Years: A Cohort Study of British Men from 1978–80 to 1998–2000

**DOI:** 10.1371/journal.pone.0019742

**Published:** 2011-05-13

**Authors:** Sheena E. Ramsay, Peter H. Whincup, Sarah L. Hardoon, Lucy T. Lennon, Richard W. Morris, S. G. Wannamethee

**Affiliations:** 1 Department of Primary Care and Population Health, University College London, London, United Kingdom; 2 Division of Community Health Sciences, St George's University of London, London, United Kingdom; University of Swansea, United Kingdom

## Abstract

**Background:**

Coronary heart disease (CHD) mortality in the UK since the late 1970s has declined more markedly among higher socioeconomic groups. However, little is known about changes in coronary risk factors in different socioeconomic groups. This study examined whether changes in established coronary risk factors in Britain over 20 years between 1978–80 and 1998–2000 differed between socioeconomic groups.

**Methods and Findings:**

A socioeconomically representative cohort of 7735 British men aged 40–59 years was followed-up from 1978–80 to 1998–2000; data on blood pressure (BP), cholesterol, body mass index (BMI) and cigarette smoking were collected at both points in 4252 survivors. Social class was based on longest-held occupation in middle-age. Compared with men in non-manual occupations, men in manual occupations experienced a greater increase in BMI (mean difference = 0.33 kg/m^2^; 95%CI 0.14–0.53; p for interaction = 0.001), a smaller decline in non-HDL cholesterol (difference in mean change = 0.18 mmol/l; 95%CI 0.11–0.25, p for interaction≤0.0001) and a smaller increase in HDL cholesterol (difference in mean change = 0.04 mmol/l; 95%CI 0.02–0.06, p for interaction≤0.0001). However, mean systolic BP declined more in manual than non-manual groups (difference in mean change = 3.6; 95%CI 2.1–5.1, p for interaction≤0.0001). The odds of being a current smoker in 1978–80 and 1998–2000 did not differ between non-manual and manual social classes (p for interaction = 0.51).

**Conclusion:**

Several key risk factors for CHD and type 2 diabetes showed less favourable changes in men in manual occupations. Continuing priority is needed to improve adverse cardiovascular risk profiles in socially disadvantaged groups in the UK.

## Introduction

Coronary heart disease (CHD) mortality and incidence has declined since the late 1970s in the UK and other developed countries [1]–[3]. The decline in CHD rates in the UK has been more marked in higher socioeconomic groups, resulting in persisting and widening inequalities in coronary disease [4], [5]. Similar observations of a lesser decline in CHD mortality in lower socioeconomic groups have been made in other Western European countries and in the USA [6], [7]. Improvements in established coronary risk factors (fall in blood pressure, cholesterol and cigarette smoking levels) are responsible for a substantial proportion of the overall fall in CHD rates [8], [9]. However, improvements in these coronary risk factors in the USA and Western Europe have not occurred uniformly in all socioeconomic groups, with more favourable reductions in adverse risk factors in higher socioeconomic groups [10]–[14]. Data from studies in the UK are limited, being based mainly on cross-sectional surveys,[15], [16] or restricted to one region [17]. Moreover, previous UK studies have provided conflicting evidence on socioeconomic trends in cigarette smoking,[15]–[18] and blood pressure [15], [17], [18]. More UK-based evidence is needed to define the extent to which risk factor trends have differed by social class. We have already shown in the British Regional Heart Study, a prospective population-based study of men from 24 towns across Britain, that relative socioeconomic differences in CHD mortality have persisted and possibly increased since the 1980s,[4] despite an overall decline in CHD mortality rates [2]. We have, therefore, investigated the patterns of changes in established coronary risk factors by socioeconomic groups in Britain over 20 years from 1978–80 to 1998–2000.

## Methods

### Study population

The British Regional Heart Study is a prospective study of cardiovascular disease comprising a socially and geographically representative sample of 7735 men initially examined in 1978–80 when aged 40–59 years, drawn from one general practice in each of 24 towns representing all major British regions [19]. At baseline a physical examination of the participants including anthropometric and physiological measurements was carried out, and blood samples were collected. Cohort participants have been followed-up since for morbidity through two-yearly reviews of general practitioner records, and for mortality through the National Health Service Central Register; contact was successfully maintained with >98% of study participants. In 1998–2000 the men, now aged 60–79 years, were invited to a 20-year re-assessment, which included completion of a questionnaire, physical examination and collection of blood samples after a minimum 6 hour fast. 4252 men (77% of surviving subjects; 80% in non-manual social classes and 70% in manual groups) attended the examination; 4094 men had at least one measurement of biological factors.

### Ethics statement

Ethical approval was provided by all relevant local research ethics committees. All participants provided written informed consent to the investigations, which were carried out in accordance with the Declaration of Helsinki.

### Socioeconomic position

Occupational social class was used as the measure of socioeconomic position. The longest-held occupation of subjects at study entry (aged 40–59 years) was used to define social class using the Registrar Generals' Social Class Classification – I (professionals, e.g. physicians, engineers), II (managerial, e.g. teachers, sales managers), III non-manual (semi-skilled non-manual, e.g. clerks, shop assistants), III manual (semi-skilled manual, e.g. bricklayers), IV (partly skilled, e.g. postmen) and V (unskilled, e.g. porters, general labourers). Information on social class was not available for 15 subjects. Men with the longest-held occupation in the armed forces were excluded from the analyses [231 at baseline (3%)].

### Coronary risk factors

Information on cigarette smoking was obtained and assessments of blood pressure, cholesterol, height and weight were carried out in the cohort at baseline (1978–80) and after 20 years (1998–2000). Detailed information on cigarette smoking was collected through questionnaires at both examinations. This was used to identify subjects who were current smokers. Physical assessments at both examinations included height and weight, measurements, and blood pressure which was measured twice in succession in the right arm with the subject seated and the arm supported. At baseline the London School of Hygiene and Tropical Medicine sphygmomanometer was used. A Dinamap 1846 oscillometric blood pressure recorder was used at the 20-year re-examination. Systolic blood pressure from the Dinamap reading was adjusted by subtracting 8 mmHg from the reading to accord with the sphygmanometer readings at baseline [20]. Blood pressure measurements at both time points were adjusted for observer variation within each town [21]. At both examinations, anthropometric measurements were made with subjects in light clothing without shoes. Height and weight were both measured while the subjects were standing. Height was measured with a Harpenden stadiometer (Critikon Service Center, Berkshire, United Kingdom) to the last complete 0.1 cm and weight with a Soehnle digital electronic scale (Critikon Service Center) to the last complete 0.1 kg. Body mass index (BMI) was calculated for each man as weight/(height)^2^ in kg/m^2^. Blood samples at baseline were used to measure serum total cholesterol measured by a modified Liebermann–Burchard method on a Technicon SMA 12/60 analyser and high-density lipoprotein (HDL) cholesterol by the Liebermann–Burchard after precipitation with magnesium phosphotungstate [22]. At the 20-year examination, serum total and HDL cholesterol were measured using a Hitachi 747 automated analyser (Hitachi, Tokyo, Japan)[23] using the Siedel[24] and Sugiuchi[25] methods respectively. The difference between total and HDL cholesterol levels was used to obtain non-HDL cholesterol levels. Total and HDL cholesterol measurements were cross-calibrated between baseline and 20 year examination using baseline residual samples [8].

### Statistical analyses

For the analyses, social classes were combined into two groups of non-manual (social class I, II, III non-manual) and manual (III manual, IV and V), with non-manual groups as the reference category. Repeated data on risk factors were available at two time points (baseline and 20 years later) for each subject. Linear regression models were constructed based on these repeated measures of age and risk factors utilising generalised estimated equations; each of BMI, systolic blood pressure, total cholesterol, and HDL and non-HDL cholesterol were regressed in turn on social class (manual versus non-manual) and time (taking the value 0 at baseline, and value 20 at 20 years), with age at that time point as a covariate to take account of increasing age of the cohort. This enabled obtaining population-averaged changes in risk factors over calendar time independent of increasing age. Estimates from the regression models were used to obtain predicted risk factors levels in 60 year old men, overall and according to social class groups, at baseline and at 20 years; this was done for the purpose of presenting secular changes in risk factors over time in a comparable age group at the two time points independent of the influences of ageing. Generalised estimating equations (GEEs) with robust standard errors were used in the regressions to take account of the repeated measures of each outcome (one at baseline and a second at 20 years) for each man. A social class*time interaction term was added to the models to assess a) whether the change in the level of the outcome (risk factor) from baseline to 20 years differed according to social class; and equivalently/correspondingly b) whether the association between the outcome (risk factor) and social class at 20 years was different from that at baseline.

Similarly, age-adjusted logistic regression with GEEs of smoking status (current versus not) on social class and time with a social class*time interaction was used to assess a) whether the odds ratio comparing social class groups at 20 years was different from that at baseline; and correspondingly b) whether the change in the odds of smoking from baseline to 20 years differed according to social class. All analyses were carried out using SAS version 9 and Stata version 10.

## Results

The proportion of non-manual and manual social classes was 41% and 59% at baseline, and 48% and 52% at the 20-year follow-up. 3324 (44%) men had died during the 20 year follow-up period (37% of non-manual and 50% of manual group); death rate was 20 per 1000 person years in non-manual groups and 28 per 1000 person years in manual groups. 4132 men alive at the 20-year follow-up with information on social class were eligible to be included in the analysis. Baseline and 20 year age-adjusted blood pressure, blood lipid and BMI levels are shown in Table 1, with corresponding age-adjusted changes, overall and separately for non-manual and manual groups. The baseline and 20-year age-adjusted mean levels presented are estimated for 60 year old men to present findings independent of the effect of increasing age. There was an overall decline in systolic blood pressure, which was more marked in men in manual social classes (−11.6 mmHg) than in men in non-manual groups (−7.9 mmHg; p for social class*time interaction <0.0001). While at baseline manual men had appreciably higher systolic blood pressure, there was little difference at follow-up. There was an overall decline in total and non-HDL cholesterol, which was more marked in non-manual men (p for interaction <0.0001 for both). While at baseline manual men had appreciably lower total and non-HDL-cholesterol, there was little difference at follow-up. There was an overall increase in HDL-cholesterol, which was more marked in non-manual men (0.19 mm/L) compared to manual men (0.15 mm/L). While at baseline HDL-cholesterol levels were similar in both groups, at follow-up HDL-cholesterol levels were more favourable among non-manual men. There was an overall increase in mean BMI, which was more marked among men in manual social classes than in men in non-manual groups (p for interaction <0.0001). Manual groups had higher mean BMI than non-manual groups at baseline (mean difference  =  0.3); this mean social class difference increased to 0.7 in 1998–2000 (p for interaction <0.0001). While at baseline manual men had a slightly higher mean BMI, this difference was considerably more marked at follow-up.

**Table 1:** Age-adjusted estimated mean levels (95%CI) of coronary risk factors in 60 year old men in manual and non-manual groups and their changes between 1978–80 and 1998–2000.

	Baseline1978–80	20 years1998–2000	Change over 20 years from baseline	p for interaction*
**Systolic blood pressure (mmHg)**				
Overall (n = 4113)	152.6 (151.7, 153.6)	142.4 (141.4, 143.4)	−10.2 (−11.9, −8.6)	
Non-manual group (n = 1955)	150.6 (149.5, 151.7)	142.6 (141.4, 143.9)	−7.9 (−9.9, −6.1)	
Manual group (n = 2158)	153.9 (152.9, 154.9)	142.3 (141.1, 143.5)	−11.6 (-13.4, −9.8)	
Manual vs. non-manual	3.3 (2.3, 4.2)	−0.4 (−1.8, 1.1)		<0.0001
**Total cholesterol (mm/L)**				
Overall (n = 3899)	6.25 (6.21, 6.30)	6.03 (5.9, 6.1)	−0.22 (−0.30, −0.14)	
Non-manual group (n = 1850)	6.36 (6.30, 6.41)	6.04 (5.98, 6.11)	−0.31 (−0.40, −0.22)	
Manual group (n = 2049)	6.19 (6.14, 6.24)	6.02 (5.95, 6.08)	−0.17 (−0.26, −0.09)	
Manual vs. non-manual	−0.17 (−0.22, −0.12)	−0.03 (−0.09, 0.04)		<0.0001
**Non-HDL cholesterol (mm/L)**				
Overall (n = 3761)	5.10 (5.01, 5.14)	4.71 (4.66, 4.76)	−0.40 (−0.48, −0.31)	
Non-manual group (n = 1783)	5.20 (5.15, 5.26)	4.69 (4.63, 4.76)	−0.51 (−0.60, −0.42)	
Manual group (n = 1978)	5.04 (4.99, 5.01)	4.71 (4.65, 4.77)	−0.33 (−0.42, −0.24)	
Manual vs. non-manual	−0.16 (−0.21, −0.11)	0.02 (−0.05, 0.09)		<0.0001
**HDL cholesterol (mm/L)**				
Overall (n = 3764)	1.15 (1.14, 1.16)	1.32 (1.30, 1.33)	0.17 (0.14, 0.19)	
Non-manual group (n = 1785)	1.15 (1.14, 1.17)	1.34 (1.32, 1.36)	0.19 (0.16, 0.21)	
Manual group (n = 1979)	1.14 (1.13, 1.16)	1.30 (1.28, 1.32)	0.15 (0.12, 0.18)	
Manual vs. non-manual	−0.01 (−0.02, 0.008)	−0.04 (−0.06, −0.02)		<0.0001
**BMI (kg/m^2^)**				
Overall (n = 4112)	25.1 (24.9, 25.3)	27.3 (27.1, 27.4)	2.2 (1.9, 2.4)	
Non-manual group (n = 1958)	24.9 (24.8, 25.1)	26.9 (26.7, 27.1)	2.0 (1.7, 2.3)	
Manual group (n = 2154)	25.2 (25.1, 25.4)	27.5 (27.4, 27.8)	2.3 (2.0, 2.6)	
Manual vs. non-manual	0.3 (0.2, 0.5)	0.7 (0.4, 0.9)		<0.0001

Analyses for each risk factor are restricted to subjects with risk factor information at both time points.

*p for interaction  =  social class*time interaction.

Table 2 presents the prevalence and change in odds of current smoking between 1978–80 and 1998–2000, and the social class difference in current smoking over this period. Overall, the odds of smoking declined over 20 years, and the extent of decline was similar in non-manual and manual groups (p for interaction 0.51). Manual social class groups had a higher level of current smokers in 1978–80 (odds ratio compared to non-manual groups = 2.24; 95%CI 2.03, 2.47), and this relative odds of smoking when comparing social classes remained unchanged after 20 years (p for interaction 0.51).

**Table 2:** Current smoking in manual compared with non-manual groups in 1978–80 and 1998–2000.

	Baseline1978–80	20 years1998–2000	Change in odds of smoking over 20 years (95% CI)	p for interaction*
Overall (n = 4121)	41%	12%	0.23 (0.19, 0.28)	
Non-manual group (n = 1959)	30%	9%	0.23 (0.19, 0.28)	
Manual group (n = 2162)	48%	16%	0.24 (0.20, 0.30)	
Odds ratio (95% CI)Manual vs. non-manual	2.24 (2.03, 2.47)	2.04 (1.68, 2.47)		0.51

*p for interaction  =  social class*time interaction.

## Discussion

In this cohort of British men, age-adjusted changes in coronary risk factors from 1978–80 resulted in persisting socioeconomic differences in these risk factors over 20 years. Marked socioeconomic differences in cigarette smoking remained unchanged since the 1980s. Although socioeconomic differences in systolic blood pressure were reduced, changes in BMI, non-HDL and HDL cholesterol were less favourable in lower socioeconomic groups.

During the past two decades, all-cause and CHD mortality rates have fallen more in non-manual social class groups, so that social inequalities in CHD mortality have widened [4]. However, the explanation for these patterns has been unclear. There are few studies in Britain with longitudinal data that report the pattern of socioeconomic changes in coronary risk factors. In western Europe and the US, socioeconomic inequalities in coronary risk factors have largely remained unchanged between the 1980s and 2000 [10]–[13]. In the present study, although cigarette smoking has declined in the population overall,[8] the degree of socioeconomic differences in cigarette smoking did not appear to change in Britain over the 20-year period from 1978–80 described in this report. This is consistent with another English study which also showed persisting socioeconomic inequalities in cigarette smoking in the late 1980s and early 1990s [18]. However, other studies found some indication of narrowing of socioeconomic differences in smoking in the UK [16], [17]. While overall levels of total cholesterol have declined in Britain between 1980 and 2000, in the present study more favourable changes in total and non-HDL cholesterol (decrease) and HDL-cholesterol levels (increase) occurred in non-manual groups. The greater decline in total and non-HDL cholesterol levels in non-manual groups is unlikely to have reflected patterns of statin use, which showed little social class difference. It may well have reflected more favourable changes in dietary saturated fat intake in non-manual groups. Although no data are available at the start of the study, at the end of the 20 year follow-up period, no marked social class differences in dietary intake of total fat, saturated and polyunsaturated fat were observed in our cohort (results not presented), consistent with the total and non-HDL cholesterol patterns observed. The more marked increase in HDL-cholesterol levels in non-manual groups, unrelated to medication use, may reflect the less marked increase in adiposity in non-manual participants. Social differences in physical activity changes are another potential explanation. However, although physical activity increased in the study population, the increase was greater in manual groups (data not presented); this pattern is unlikely to explain the observed changes in HDL-cholesterol. The significantly greater decline in blood pressure in manual social groups, which greatly reduced socioeconomic differences in systolic blood pressure, was consistent with previous studies from the UK [15], [17] and the US [12]. Although blood pressure lowering medications made a major contribution to the overall decline in blood pressure in this cohort [26], there was no social class difference in the use of blood pressure lowering medications in this study and sensitivity analyses excluding those on blood pressure lowering treatment did not affect the results. The reason for the greater decline in blood pressure lowering treatment in manual men, therefore, remains uncertain. Increasing physical activity [8], particularly among manual men, could have contributed; changes in diet could also be important. The greater increases in BMI in manual men, with markedly higher BMI levels in manual men at the end of the follow-up period, are consistent with results from the Whitehall II study, which observed that marked socioeconomic gradients in BMI were present in the 1980s and 1990s [18], although a study in north west England reported a narrowing of socioeconomic inequalities in BMI and blood pressure during the 1990s [17]. These changes are not likely to have been explained by changes in physical activity (with increases being seen particularly among manual men), raising the possibility that adverse changes in diet, particularly among manual men, may have been responsible.

### Strengths and weaknesses

This study presents important data on changes in coronary risk factors according to socioeconomic groups in a large socially and geographically representative British cohort. Contact has been maintained with more than 98% of the cohort since initial recruitment in 1978–80. Socioeconomic position in the present study was based on the longest-held occupation of the subjects assessed at middle-age for almost all participants. Social class changed only for a small proportion of subjects (<10%) over the 20-year period investigated in this paper. An inevitable result of using a cohort study is the restriction of analyses to subjects who were alive at the end of the 20-year follow-up period. Survivors are likely to have a better cardiovascular risk profile, consequently, within each socioeconomic group favourable changes over 20 years in risk factors (systolic BP) may have been overestimated and unfavourable changes in risk factors (BMI, HDL cholesterol) may have been underestimated. However, importantly, since these changes within each socioeconomic group will have been affected in the same way, the trends in the *differences* in risk factors between socioeconomic groups, which constitute the key findings of the study, are unlikely to be biased. A strength of the study is the high response rate at follow-up (77%), which minimises response bias. Although non-response was slightly greater among manual compared to non-manual groups, it is unlikely to bias the trends in social class differences in risk factors. Levels of BMI and cholesterol were similar in responders and non-responders of the 20-year follow-up examination of the cohort, although smoking levels were greater in non-responders [27]. However, again, while this may have led to overestimation of the decline in smoking within each socioeconomic group, it is unlikely to impact on the trends in the risk factor differences between the groups. A possible limitation is that since the study is based on men and excluded towns with high mobility, the results may not be generalisable to women and to ethnic minority populations. We acknowledge that when studying risk factor changes over a period of time, time trends and age-related changes are closely related. Despite the methods used to present age-adjusted secular changes in risk factors, the effect of increasing age on risk factors may not have fully been taken into account and we cannot rule out the possibility of some residual effect of ageing on the observed results. Furthermore, since the results are based on middle-aged British subjects (40–59 years) in 1978–80, the findings may not necessarily be extrapolated to other generations. A limitation of the present study is that explanations of observed socioeconomic changes in risk factors were not explored; possible factors include diet and other lifestyle changes and this issue merits further investigation. However, the main strength of this paper is that it presents socioeconomic trends in major coronary risk factors in Britain using longitudinal data in a well-defined population over a period of time using a stable marker of socioeconomic position; this is in contrast to previous British studies based on cross-sectional surveys [15], [16].

### Implications and conclusions

The results of this study imply that although socioeconomic differences in systolic blood pressure have narrowed over 20 years since 1980, inequalities in other major coronary risk factors including cigarette smoking have persisted, while inequalities in BMI and HDL-cholesterol may be increasing. These secular trends in socioeconomic differences in coronary risk factors (independent of increasing age of our cohort) reflect the unchanging or perhaps increasing social inequalities in CHD occurring in Britain [4], [28]. Increasing socioeconomic differences in BMI, in particular, are likely to lead to a further widening of inequalities in CHD and other chronic diseases, particularly type 2 diabetes. Despite the high priority given to reducing inequalities in CHD in national policies,[28] unfavourable socioeconomic trends in major risk factors (BMI, HDL-cholesterol and cigarette smoking) appear to be occurring. If measures are not taken to address these unfavourable trends, inequalities in coronary risk factors will continue to widen. Therefore, there is an increasing need for evidence-based public health interventions focussing on improving diet and physical activity and their implementation to improve coronary risk factors levels in the population with a specific focus on reducing existing inequalities in these risk factors.

## References

[1] Allender S, Peto V, Scarborough P, Boxer A, Rayner M (2008). Coronary heart disease statistics 2008..

[2] Lampe FC, Morris RW, Walker M, Shaper AG, Whincup PH (2005). Trends in rates of different forms of diagnosed coronary heart disease, 1978 to 2000: prospective, population based study of British men.. BMJ.

[3] Ford ES, Ajani UA, Croft JB, Critchley JA, Labarthe DR (2007). Explaining the Decrease in U.S. Deaths from Coronary Disease, 1980-2000.. N Engl J Med.

[4] Ramsay SE, Morris RW, Whincup PH, Lennon LT, Wannamethee SG (2008). Are social inequalities in mortality in Britain narrowing? Time trends from 1978 to 2005 in a population-based study of older men.. J Epidemiol Community Health.

[5] Acheson D (1998). Independent Inquiry into Inequalities in Health..

[6] Mackenbach JP, Bos V, Andersen O, Cardano M, Costa G (2003). Widening socioeconomic inequalities in mortality in six Western European countries.. Int J Epidemiol.

[7] Steenland K, Henley J, Thun M (2002). All-Cause and Cause-specific Death Rates by Educational Status for Two Million People in Two American Cancer Society Cohorts, 1959-1996.. Am J Epidemiol.

[8] Hardoon SL, Whincup PH, Lennon LT, Wannamethee SG, Capewell S (2008). How much of the recent decline in the incidence of myocardial infarction in British men can be explained by changes in cardiovascular risk factors? Evidence from a prospective population-based study.. Circulation.

[9] Unal B, Critchley JA, Capewell S (2004). Explaining the Decline in Coronary Heart Disease Mortality in England and Wales Between 1981 and 2000.. Circulation.

[10] Galobardes B, Costanza MC, Bernstein MS, Delhumeau C, Morabia A (2003). Trends in Risk Factors for Lifestyle-Related Diseases by Socioeconomic Position in Geneva, Switzerland, 1993-2000: Health Inequalities Persist.. Am J Public Health.

[11] Iribarren C, Luepker RV, McGovern PG, Arnett DK, Blackburn H (1997). Twelve-Year Trends in Cardiovascular Disease Risk Factors in the Minnesota Heart Survey: Are Socioeconomic Differences Widening?. Arch Intern Med.

[12] Kanjilal S, Gregg EW, Cheng YJ, Zhang P, Nelson DE (2006). Socioeconomic Status and Trends in Disparities in 4 Major Risk Factors for Cardiovascular Disease Among US Adults, 1971-2002.. Arch Intern Med.

[13] Osler M, Gerdes LU, Davidsen M, Bronnum-Hansen H, Madsen M (2000). Socioeconomic status and trends in risk factors for cardiovascular diseases in the Danish MONICA population, 1982-1992.. J Epidemiol Community Health.

[14] Peltonen M, Huhtasaari F, Stegmayr B, Lundberg V, Asplund K (1998). Secular trends in social patterning of cardiovascular risk factor levels in Sweden. The Northern Sweden MONICA Study 1986-1994. Multinational Monitoring of Trends and Determinants in Cardiovascular Disease.. J Intern Med.

[15] Bartley M, Fitzpatrick R, Firth D, Marmot M (2000). Social distribution of cardiovascular disease risk factors: change among men in England 1984-1993.. J Epidemiol Community Health.

[16] Giskes K, Kunst AE, Benach J, Borrell C, Costa G (2005). Trends in smoking behaviour between 1985 and 2000 in nine European countries by education.. J Epidemiol Community Health.

[17] Lyratzopoulos G, Heller RF, McElduff P, Hanily M, Lewis P (2006). Deprivation and trends in blood pressure, cholesterol, body mass index and smoking among participants of a UK primary care-based cardiovascular risk factor screening programme: both narrowing and widening in cardiovascular risk factor inequalities.. Heart.

[18] Ferrie JE, Shipley MJ, Davey Smith G, Stansfeld SA, Marmot MG (2002). Change in health inequalities among British civil servants: the Whitehall II study.. J Epidemiol Community Health.

[19] Walker M, Whincup PH, Shaper AG (2004). The British Regional Heart Study 1975-2004.. Int J Epidemiol.

[20] Whincup PH, Bruce NG, Cook DG, Shaper AG (1992). The Dinamap 1846SX automated blood pressure recorder: comparison with the Hawksley random zero sphygmomanometer under field conditions.. J Epidemiol Community Health.

[21] Bruce NG, Cook DG, Shaper AG (1990). Differences between observers in blood pressure measurement with an automatic oscillometric recorder.. J Hypertens.

[22] Thelle DS, Shaper AG, Whitehead TP, Bullock DG, Ashby D (1983). Blood lipids in middle-aged British men.. Br Heart J.

[23] Emberson J, Whincup PH, Walker M, Thomas M, Alberti KGMM (2002). Biochemical measures in a population-based study: effect of fasting duration and time of day.. Ann Clin Biochem.

[24] Siedel J, Hagele EO, Ziegenhorn J, Wahlefield AW (1983). Reagent for the enzymatic determination of serum total with improved lipolytic efficiency.. Clin Chem.

[25] Sugiuchi H, Uji Y, Okabe H, Irie T, Uekama K (1995). Direct measurement of high-density lipoprotein cholesterol in serum with polyethylene glycol-modified enzymes and sulfated alpha-cyclodextrin.. Clin Chem.

[26] Hardoon SL, Whincup PH, Wannamethee SG, Lennon LT, Capewell S (2010). Assessing the impact of medication use on trends in major coronary risk factors in older British men: a cohort study.. Eur J Cardiovasc Prev Rehabil.

[27] Thomas MC, Walker M, Lennon LT, Thomson AG, Lampe FC (2002). Non-attendance at re-examination 20 years after screening in the British Regional Heart Study.. J Public Health.

[28] Department of Health (2005). Tackling Health Inequalities: Status Report on the Programme for Action..

